# 

**Published:** 1898-12

**Authors:** 


### CONTENTS.

COMMUNICATIONS.

Report of the Committee on Foreign Relations of the National Association of Dental Faculties........................................ ........ 551

PROCEEDINGS.

Tri-State Meeting.................................................................... 566

"Degeneration and Decay of Oral Tissues from Lack of Exercise," 574

EDITORIAL.

The National Dental Technic Association.................................. 591

Close of the Year...................................................................... 592

